# Flourishing Privately but Languishing Publicly: Ethnic Identity’s Contribution to Understanding Eudaimonic Wellbeing

**DOI:** 10.3390/ijerph192114156

**Published:** 2022-10-29

**Authors:** Mary J. Arneaud, Nicole Alea, Theodore E. A. Waters

**Affiliations:** 1 School of Social Sciences, Heriot-Watt University Dubai, Dubai P.O. Box 38103, United Arab Emirates; 2 Psychological & Brain Sciences, University of California, Santa Barbara, CA 93106, USA; 3 Division of Science, New York University Abu Dhabi, Abu Dhabi P.O. Box 129188, United Arab Emirates

**Keywords:** eudaimonic wellbeing, positive relations with others, social integration, ethnic identity, ethnic diversity

## Abstract

The paper probes the meaning of wellbeing by examining whether ethnic identity is related to private and public conceptualisations of eudaimonic wellbeing. Private and public eudaimonic wellbeing are assessed as positive relations with others and social integration. Ethnic identity is a type of social identity that is meaningful in contexts of enduring inter-ethnic group contact. Fiji and Trinidad and Tobago (TT), nations with contact between two major ethnic groups for over a century, are the contexts for a preliminary exploration. Young adults (Fiji *N* = 38, 19–26 years old; TT *N* = 41, 18–25 years old) completed measures of positive relations with others (private eudaimonic wellbeing), social integration (public eudaimonic wellbeing), and ethnic identity development. Across the nations, a stronger sense of ethnic identity, or commitment to the ingroup, predicted better positive relations with others but worse social integration. Ethnic identity thus seems to be a key construct in understanding positive private, but negative public eudaimonic wellbeing among young adults in contexts of ethnic diversity. Findings are discussed by considering how implications of ethnic diversity (competitive inter-group relations, inter-group contact making ethnic group membership salient) might be related to ethnic identity development, and private and public eudaimonic wellbeing.

## 1. Introduction

The purpose of the current study is to probe the meaning of private and public conceptualisations of eudaimonic wellbeing by examining their relations with ethnic identity (cf. [1,2,3,4]). Eudaimonic wellbeing has its conceptual foundations in psychological and sociological theories [3,5], and provides a comprehensive approach to understanding wellbeing in people’s private (psychological) and public (social) lives (cf. [1]). Eudaimonic wellbeing is about how successfully or unsuccessfully people navigate life’s challenges, thereby developing or not developing their full potentials [3]. *Eudaimonia*, translated from Greek, means “good or healthy” (*eu*) “true self” (*daimon*; [6], p. 6); thus, both wellbeing and identity are integral constructs in describing and explaining eudaimonia.

*Ethnic* identity, specifically, is a meaningful construct in contexts where two or more ethnic groups have been in contact over a period of time [7]. People around the world perceive that their societies have become more ethnically diverse in the past 20 years [8]. Ethnic identity thus provides a contemporary lens through which to further understand eudaimonic wellbeing. The construct is about developing a sense of belonging to an individual’s ethnic ingroup (e.g., Asian) by engaging in two distinct but strongly related processes, exploration and commitment [9]. The exploration process involves considering ingroup ethnic identity alternatives, for example, by reading, discussing, and participating in cultural activities [10]. After exploration, decisions or commitments to choices about what to believe (e.g., religion) and value (e.g., political ideology), and how to behave (e.g., language usage) can be made [7,10], and a sense of ingroup belonging, or ethnic identity, developed [9].

We seek to better understand what private and public eudaimonic wellbeing mean in relation to ethnic identity. We therefore draw from two multidimensional models of psychological [11] and social [5] wellbeing. The models provide detailed descriptions of the private and public criteria individuals can use to evaluate whether they are flourishing or languishing in the face of life’s private and public challenges (cf. [1]). However, we focus only on those dimensions that are conceptually similar to ethnic identity: *positive relations with others* from the model of psychological wellbeing and *social integration* from the model of social wellbeing.

Positive relations with others is about functioning positively in one’s private life by developing and maintaining warm and trusting interpersonal relationships [3]. Higher levels of positive relations with others is characterised as flourishing, whereas lower levels is described as languishing (cf. [1]). Positive relations with others is conceptually similar to ethnic identity insofar as ethnic identity development is situated in interactions with others ([12]; e.g., discussions, participation in cultural activities). A meta-analysis of the relation between a component of ethnic identity commitment (positive ethnic–racial affect) and wellbeing among diverse ethnic minority group samples in the US showed that, compared with other indicators of wellbeing (e.g., self-esteem), the strongest—and a positive—relation was with positive social functioning (i.e., [13]).

Social integration is about functioning well in one’s public life by feeling that one belongs to and is accepted by their communities [1]. Higher levels of social integration is characterised as flourishing, whereas lower levels is described as languishing (cf. [1]). Social integration is conceptually similar to ethnic identity as ethnic identity is also about developing a sense of belonging to others–although these others are specifically members of an individual’s ethnic *in*group, versus every member of the community. Contexts of ethnic diversity–where ethnic identity is meaningful [7]–include members of ethnic groups to which the individual does not belong, or ethnic *out*groups. Thus, for example, for an individual who identifies as Asian (*in*group), living in an ethnically diverse community where there are those who identify as members of other ethnic groups (*out*groups), flourishing in public life is about feelings of belonging to and acceptance by other Asians *and* the members of the other ethnic groups in the community, whereas languishing is about feeling disconnected.

There seems to be less research examining relations between ethnic identity and indicators of public eudaimonic wellbeing. Further, studies which examined a construct similar to social integration, perceived societal wellbeing, showed somewhat mixed results [14,15]. For example, a construct similar to ethnic identity commitment (group membership evaluation) was unrelated to perceived societal wellbeing in one sample [14], but positively related in others [14,15], though both relations were relatively small in magnitude.

Examining the relation between ethnic identity and social integration might be a particularly important for better understanding public eudaimonic wellbeing in ethnically diverse contexts. Individuals might not experience feelings of belonging and acceptance (social integration) among those members of the community from ethnic outgroups. Thus, developing a sense of belonging to one’s ethnic ingroup (ethnic identity), which is typically beneficial for various indicators of wellbeing (e.g., [13,16]), might actually be costly for social integration.

We thus explore private (positive relations with others) and public (social integration) eudaimonic wellbeing at a stage in life when wellbeing is particularly related to ethnic identity development–the late teens to the mid-twenties (e.g., [16]). Our samples are from societies where there are two major ethnic groups that have been in contact for over a century (cf. [7]), Fiji and Trinidad and Tobago (TT). In Fiji, there is an indigenous group, the iTaukei, that constitutes 56.82% of the population, and an Indian-descended group (henceforth, Indo-Fijians), which constitutes 37.48% [17]. In TT there is an African-descended group (Afro-Trinbagonians), that constitutes 34.22% of the population, and an Indian-descended group (Indo-Trinbagonians), which constitute 35.43% (Ministry of Planning and Sustainable Development, 2011 [18]). 

Socio-economic status might be related to indicators of eudaimonic wellbeing (cf. [5]). In Fiji and TT, however, inequality seems to be more variable *within* rather than *between* the major ethnic groups. For example, the average income of the iTaukei is slightly lower than of Indo-Fijians, but Indo-Fijians are overrepresented among the poor [19]. Comparably, in TT, aggregate ethnic educational and occupational segregation is low, which indicates that in general, neither of the major ethnic groups is either over- or under-represented in educational status or certain occupations [20].

The current contexts are thus different from the majority-minority contexts of extant research (e.g., [13,14,15]). However, the relative equivalence regarding number and socio-economic status between the major ethnic groups might be especially instructive for understanding private, and perhaps particularly, public eudaimonic wellbeing in relation to ethnic identity. For example, in order to understand the precise relation between context and ethnic identity, empirical research among young people who are not developing in countries where they are ethnic minorities (e.g., Trinidad) has been recommended (e.g., [21]). However, to the best of our knowledge, until now this work has not been conducted in TT. In Fiji, a concept similar to ethnic identity commitment, in-group identification, has been studied [22], but not in relation to wellbeing.

Three specific hypotheses are explored in the current study. First, for private eudaimonic wellbeing, similar to extant research (e.g., [13]), it is expected that higher levels of the processes of ethnic identity development, exploration and commitment, will predict higher levels of positive relations with others. Second, for public eudaimonic wellbeing, we consider the ways ethnic identity and social integration are simultaneously conceptually similar and different, and the current research contexts. And, so, different from extant research [14,15], it is expected that higher levels of exploration and commitment will predict lower levels of social integration. Finally, although the nations are similar in ways that are important for the relation between ethnic identity and eudaimonic wellbeing, they are also obviously different societies. We therefore explore whether the relations between exploration and commitment and private and public eudaimonic wellbeing will differ across the nations.

## 2. Method

### 2.1. Participants

Seventy-nine young adults from Fiji and TT participated. Participants from Fiji (*N* = 38; *M* age = 22.03, *SD* = 1.62; female = 42.1%) did not differ from participants from TT (*N* = 41; *M* age = 21.27, *SD* = 1.94; female = 48.8%) by age, *t* (77) = −1.88, *p* = 0.064, or sex, *χ*^2^ (1) = 0.354, *p* = 0.552. The ethnic distributions of the samples were relatively comparable to the ethnic compositions of each nation. Fiji ethnic groups included: iTaukei = 42.1%, Indo-Fijian = 42.1%, and other = 15.8%. TT ethnic groups included: Afro-Trinbagonian = 29.3%, Indo-Trinbagonian = 39%, mixed = 29.3%, other = 2.4%. In Fiji participants were recruited via an email to a university listserv for Fijian nationals, social media, and snowball sampling in 2015. In TT, participants were recruited from undergraduate psychology courses at a university, notice boards on campus, and social media from 2012 to 2014. Participants in Fiji were compensated with the local currency equivalent of USD 10.00. Participants in TT from psychology courses were compensated with partial credit towards a research requirement; non-psychology students received the local currency equivalent of USD 15.00.

### 2.2. Measures and Procedures

For both samples, data were collected as parts of larger studies, and took place in university settings (e.g., offices, small conference rooms) following informed consent (details about the other measures completed for both studies are available from the first author). In Fiji, participants independently completed written questionnaires in small group settings. Ethnic identity was measured before eudaimonic wellbeing. In TT, data were collected via structured, oral, one-on-one interviews. The ethnic identity and eudaimonic wellbeing measures were counterbalanced. In both nations, the eudaimonic wellbeing measures were always given together, with private preceding public. The measures for the current study took 15 to 20 min to complete.

#### 2.2.1. Private Eudaimonic Wellbeing: Positive Relations with Others

Private eudaimonic wellbeing was assessed with nine items from the positive relations with others subscale of the Scales of Psychological Wellbeing [11]. Individuals evaluate their feelings, perceptions, and abilities about maintaining warm, satisfying, and trusting relationships. The subscale assesses how individuals think about their adjustment in relation to immediate others (e.g., *I don’t have many people who want to listen when I need to talk*), and their perceptions of others’ outlook on them as a friend or family member (e.g., *I know that I can trust my friends, and they know they can trust me*). Responses are made on a six-point Likert scale ranging from *strongly disagree* to *strongly agree*, with higher scores indicating greater agreement that one’s interpersonal relationship are positive. Cronbach’s alphas were Fiji = 0.70 and TT = 0.69.

#### 2.2.2. Public Eudaimonic Wellbeing: Social Integration

Public eudaimonic wellbeing was assessed with seven items from the social integration subscale of the Scales of Social Wellbeing [5]. Individuals evaluate their feelings and perceptions about having a sense of belonging and connection to others in the community and society. The subscale assesses how individuals think about their adjustment in relation to others in their communities (e.g., *You don’t feel you belong to anything you’d call a community*), and their perceptions of other’s outlook on them as a member of the society (e.g., *You believe other people in society value you as a person*). Participants from Fiji responded to the original measure’s second-person pronoun items, whereas Trinbagonian participants responded to the modified measure’s first-person pronoun items (e.g., *I feel close to other people in my community*; (cf. [5])). Responses are made on a six-point Likert scale ranging from *disagree strongly* to *agree strongly*, with higher scores indicating a stronger sense of social integration. Cronbach’s alphas were Fiji = 0.76 and TT = 0.87.

#### 2.2.3. Ethnic Identity Development: Exploration and Commitment

Ethnic identity was assessed with the 12-item Revised-Multigroup Ethnic Identity Measure (Revised-MEIM; [9]). Individuals evaluate their exploration of (five items, e.g., I think a lot about how my life will be affected by my ethnic group membership) and commitment to (seven items, e.g., I feel good about my cultural or ethnic background) ethnic identity choices. Participants from Fiji responded to the original measure’s four-point Likert scale ranging from strongly disagree to strongly agree, whereas those from TT responded to a modified five-point Likert scale ranging from strongly disagree to strongly agree. In both cases, higher scores indicate greater agreement that one has considered and made decisions about ethnic identity choices. Based on previous work that also used the Revised-MEIM with four-point and five-point Likert scales across two different samples (i.e., [23]), we equated the response scales by recoding the values to range from 0–1 (i.e., Fiji: 1 = 0.25, 2 = 0.50, 3 = 0.75, 4 = 1; TT: 1 = 0.20, 2 = 0.40, 3 = 0.60, 4 = 0.80, 5 = 1). Cronbach’s alpha values were exploration-Fiji = 0.78, TT = 0.62, commitment–Fiji = 0.92, TT = 0.80.

## 3. Results

### 3.1. Preliminary Analyses

Initial correlational analyses explored bivariate associations between background variables (age, sex), nation, ethnic identity (exploration and commitment), and private (positive relations with others) and public (social integration) eudaimonic wellbeing (see Table 1). Age and sex were not related to ethnic identity or eudaimonic wellbeing, and are not considered further. Identical to previous research, the ethnic identity exploration and commitment processes were strongly positively related. Additionally, participants were more likely to be committed to than exploring their ethnic identities, *t* (79) = −6.498, *p* < 0.001, *d* = 0.732. The two eudaimonic wellbeing variables, positive relations with others and social integration, were negatively related, supporting the distinction between private and public dimensions of eudaimonic wellbeing [1]. Levels of private eudaimonic wellbeing (positive relations with others) were also higher than public eudaimonic wellbeing (social integration), *t* (78) = 8.718, *p* < 0.001, *d* = 0.987. Finally, participants from Fiji were more likely to be engaging in the exploration and commitment processes than those from TT.

### 3.2. Ethnic Identity and Eudaimonic Wellbeing

Relations between ethnic identity, private and public eudaimonic wellbeing, considering nation, corresponding to study hypotheses, were explored with hierarchical moderation regression analyses. Two analyses were conducted for each outcome variable: one with private eudaimonic wellbeing—positive relations with others—and the other with public eudaimonic wellbeing—social integration. Ethnic identity predictors were exploration and commitment, and entered together in the first step of the regression model (this was also the reason that the moderation PROCESS macro [24] was not used for analyses; it does not allow for multiple predictors to be included in moderation analyses simultaneously). Nation was entered in the second step, and the interaction terms in the third step. The interaction terms used were unstandardized residuals of exploration by nation, and commitment by nation to avoid multicollinearity of the interaction terms with the effects of these variables entered in steps one and two. Assumptions were met, but due to the relatively small sample size bootstrapping (1000 samples, 95% confidence interval) was used. Standardized beta weights (*β*) are reported.

#### 3.2.1. Ethnic Identity and Private Eudaimonic Wellbeing

Results from the regression analyses for private eudaimonic wellbeing, or positive relations with others as the outcome variable, are in Table 2. The two ethnic identity processes, exploration and commitment, together accounted for 8.0% of the variance in positive relations with others, *R*^2^ = 0.080, *F* (2, 75) = 3.242, *p* = 0.045. As seen from the *β*-weights, and as expected, ethnic identity commitment was a significant predictor of positive relations with others: higher levels of commitment were associated with better private interpersonal relationships. There was no association between ethnic identity exploration and positive relations with others when one’s nation is not considered. Including nation in the model only added 1% additional explained variance, which was not significant, *R*^2^ = 0.089, Δ*R*^2^ = 0.010, Δ*F* (1, 74) = 0.805, *p* = 0.373. The interaction terms added an additional 6.6% of variance to the model. However, this was only marginally significant, *R*^2^ = 0.155, Δ*R*^2^ = 0.066, Δ*F* (2, 72) = 2.809, *p* = 0.067; therefore, the effect was not explored further.

#### 3.2.2. Ethnic Identity and Public Eudaimonic Wellbeing

Results from the analyses for public eudaimonic wellbeing, or social integration, are also in Table 2. Exploration and commitment together accounted for 24.3% of the variance in social integration, *R*^2^ = 0.243, *F* (2, 76) = 12.213, *p* < 0.001. The *β*-weights indicate that, as expected, ethnic identity commitment, although not exploration, was a significant predictor of social integration. The pattern was as hypothesised: the more someone was committed to their ethnic ingroup, the less they felt a sense of social integration or public eudaimonic wellbeing. Including nation in the second step added an additional 3.51% of variance to the model. However, this addition was only marginally significant, *R*^2^ = 0.279, Δ*R*^2^ = 0.035, Δ*F* (1, 75) = 3.671, *p* = 0.059, and therefore not interpreted further. The nation interactions with either exploration or commitment did not add significant additional explained variance, *R*^2^ = 0.289, Δ*R*^2^ = 0.010, Δ*F* (2, 73) = 0.521, *p* = 0.596.

## 4. Discussion

### 4.1. Flourishing Privately: Ethnic Identity’s Contribution to Understanding Positive Relations with Others

Across the nations, ethnic identity commitment was associated with better interpersonal relationships. This finding can perhaps be understood by considering results from the current study’s preliminary analyses along with an implication of ethnic diversity. Preliminary analyses indicated that participants were more likely to have made decisions about their beliefs, values, and behaviours, or committed to their ethnic identities, than to be considering or exploring these alternatives. Additionally, levels of positive relations with others were higher than levels of social integration. Like other contexts characterised by ethnic diversity, the current contexts are described as having competitive inter-group relations over access to resources [25,26,27]. For example, the members of each of the major ethnic groups perceive that their ingroup dominates one—but not the other—major arena of power within the societies, as members of the iTaukei and Afro-Trinbagonian groups form their nation’s political elite, whereas Indo-Fijians and Indo-Trinbagonians are among their nation’s economic elite [25,26]. Ethnic tensions seem to be related to making strong commitments to ingroup beliefs, values (e.g., political ideology), and behaviours (cf. [27]). Consistent with the higher levels of positive relations with others than social integration found, ethnic tensions might also be related to developing and maintaining warm and trusting relationships with family members and friends (higher positive relations with others), who might be mostly from an individual’s ethnic ingroup, as a way of successfully navigating the challenges of living in contexts of ethnic diversity (cf. [28]). Thus, it makes sense that in ethnically-diverse contexts developing a sense of ingroup belonging seems to be, at least in part (only 8% of variance was explained), key to flourishing in one’s private life by having warm, satisfying, and trusting relationships interpersonal relationships.

### 4.2. Languishing Publicly: Ethnic Identity’s Contribution to Understanding Social Integration

Across the nations, ethnic identity commitment was also associated with worse social integration. This finding can also perhaps be understood by keeping the results of the current preliminary analyses together with another implication of ethnic diversity in mind. Again, participants were more likely to be committed to than exploring their ethnic identities. Additionally, however, social integration and positive relations with others were negatively related. Contexts of ethnic diversity are those where individuals belong to groups that differ by factors, such as place of origin, religious affiliation and practice, and cultural activities and consumption (e.g., food, music and dance, traditional garments, etc.; (cf. [7])). The inter-group contact provided by ethnically diverse contexts, in which individuals come face-to-face with others having different beliefs and behaviours, makes ethnic group membership salient, which leads to exploration [29] and promotes commitment [10]. However, consistent with the negative relation found between social integration and positive relations with others, contexts of ethnic diversity might also be related to feeling detached from the community (lower social integration), which includes different others. It thus also makes sense that in ethnically-diverse contexts developing a sense of ingroup belonging seems to be in part (almost a quarter of variance explained) key to languishing in one’s public life by feeling detached from others in one’s community.

## 5. Conclusions

This study was obviously limited in terms of modest *N*, convenience samples of university students, and measure and procedural equivalence across the two samples. However, we did not further explore or interpret marginally significant cross-national effects. The links are also correlational. It is possible that private and public eudaimonic wellbeing precede ethnic identity development. Thus, longitudinal developmental and experimental studies are needed. Even so, our preliminary exploration contributes to clarifying what eudaimonic wellbeing might be for young adults in contexts of ethnic diversity. Developing a sense of belonging to the ethnic ingroup (ethnic identity commitment) helps us to understand positive private (developing and maintaining warm and trusting interpersonal relationships), but negative public (feeling detached from the community) eudaimonic wellbeing. Thus, we are left with the question of how to develop a sense of ethnic ingroup belonging and simultaneously flourish in private and public life. One avenue might be via involvement with communities that include members of ethnic outgroups. Social integration is positively associated with having worked with others in the community to solve a problem in the past 12 months [5]. Thus, future research might investigate whether recent prosocial activity with a community of diverse others, might moderate the relations between ethnic identity commitment and both positive relations with others and social integration among young adults from ethnically diverse contexts.

## Figures and Tables

**Table 1:** Descriptive statistics and correlations for study variables.

Variable	*M* (*SD*)	1	2	3	4	5	6	7
Age	21.63 (1.82)	--						
2. Sex	--	−0.144	--					
3. Nation	--	0.209	0.067	--				
4. Exploration	0.656 (0.159)	0.117	−0.043	0.288 *	--			
5. Commitment	0.750 (0.136)	0.058	−0.100	0.290 **	0.629 **	--		
6. Positive relations	4.50 (0.682)	0.115	0.055	0.168	0.145	0.280 *	--	
7. Social integration	3.09 (0.988)	−0.091	−0.147	−0.327 **	−0.344 **	−0.491 **	−0.502 **	--

* *p* < 0.05, ** *p* < 0.001. Note. Sex: 1 = female, 2 = male; Nation: 1 = TT, 2 = Fiji; Ethnic identity range: 0 to 1; Eudaimonic wellbeing range: 1 to 6.

**Table 2:** Moderator analysis: dimensions of private and public eudaimonic wellbeing.

	Positive Relations			Social Integration		
	Δ*R*^2^	*β*	*t*	Δ*R*^2^	*β*	*t*
Step 1	0.080 *			0.243 ***		
Exploration		−0.047	−0.333		−0.058	−0.453
Commitment		0.309	2.184 *		−0.455	−3.542 ***
Step 2	0.010			0.035 ^		
Nation		0.105	0.897		−0.198	−1.916 ^
Step 3	0.066 ^			0.010		
Exploration × Nation		−0.332	−2.369 *		0.129	1.005
Commitment × Nation		0.205	1.485		−0.059	−0.470

^ *p* < 0.10; * *p* < 0.05; ***p < 0.001. Note. Nation: 1 = TT, 2 = Fijian. The pattern of previous statistically significant effects remained consistent at each new step of the model.

## Data Availability

The data set is available upon request.
